# A Case Report of SARS-CoV-2 and Salmonella Coinfection in an Infant With Brain Abscess and Sepsis

**DOI:** 10.7759/cureus.46320

**Published:** 2023-10-01

**Authors:** Amal Aldhaheri, Sarah Nagadi, Abeer Alnajjar, Khouloud Alsofyani, Mohammad S Agha

**Affiliations:** 1 Pediatric Infectious Diseases, Faculty of Medicine, King Abdulaziz University, Jeddah, SAU; 2 Pediatrics, Faculty of Medicine, King Abdulaziz University, Jeddah, SAU; 3 Pediatrics, National Research Center, Cairo, EGY

**Keywords:** infant, brain abscess, sepsis, covid-19, salmonella

## Abstract

The impact of community-acquired bacteremia on the prognosis of children with COVID-19 is an area of ongoing research. Pediatric data on this aspect is limited. Here, we report the case of a four-month-old male infant who presented to King Abdulaziz University Hospital in January 2022 with a co-infection of COVID-19 and *Salmonella *meningitis, and sepsis complicated by a brain abscess.

## Introduction

The outbreak of the SARS-CoV-2 infection occurred in December 2019 in Wuhan City, China. Since then, the virus has spread to several countries. Published data from numerous countries reveals that pediatric patients represent a minor proportion of COVID-19 cases, accounting for <2% of all the cases [1]. In addition, these patients manifest less prominent clinical symptoms and a lower fatality rate than those of the adult population [1]. Adverse outcomes in COVID-19 patients are not always due to the direct impact of the viral infection but are often due to bacterial co-infections [2]. In this article, we report the case of a four-month-old male infant who presented to King Abdulaziz University Hospital in January 2022 with a co-infection of COVID-19 and *Salmonella* meningitis with sepsis complicated by a brain abscess. 

## Case presentation

A four-month-old male infant presented to the emergency department on January 4, 2022, with a three-day history of fever and abnormal movements. The patient was born at term by spontaneous vaginal delivery with a birth weight of 3 kg. His vaccinations were up to date, and his developmental milestones were appropriate for his age.

The patient was healthy up to one week prior to admission; at that time, he developed persistent spikes of fever in the range of 38°C to 39°C, which subsided with the administration of antipyretics. There was an associated productive cough and post-tussive vomiting of thick green sputum. Moreover, there was a history of positive contact with a patient who suffered from an infection of the upper respiratory tract and four episodes of loose stools, which were moderate in amount and non-bloody. One day prior to admission, the patient developed seizures in the form of generalized tonic-clonic movements with up-rolling of the eyes. Some incidents lasted for approximately 10 minutes.

On his presentation to the emergency department, the patient appeared ill. His vital signs were as follows: temperature 39°C; heart rate 179 beats/min; respiratory rate 40 breaths/min; blood pressure (systolic/diastolic) 98/58 mm Hg; and oxygen saturation on room air 95%. His growth parameters were as follows: weight 7 kg; length 64 cm; and head circumference 42 cm.

**Figure 1:**
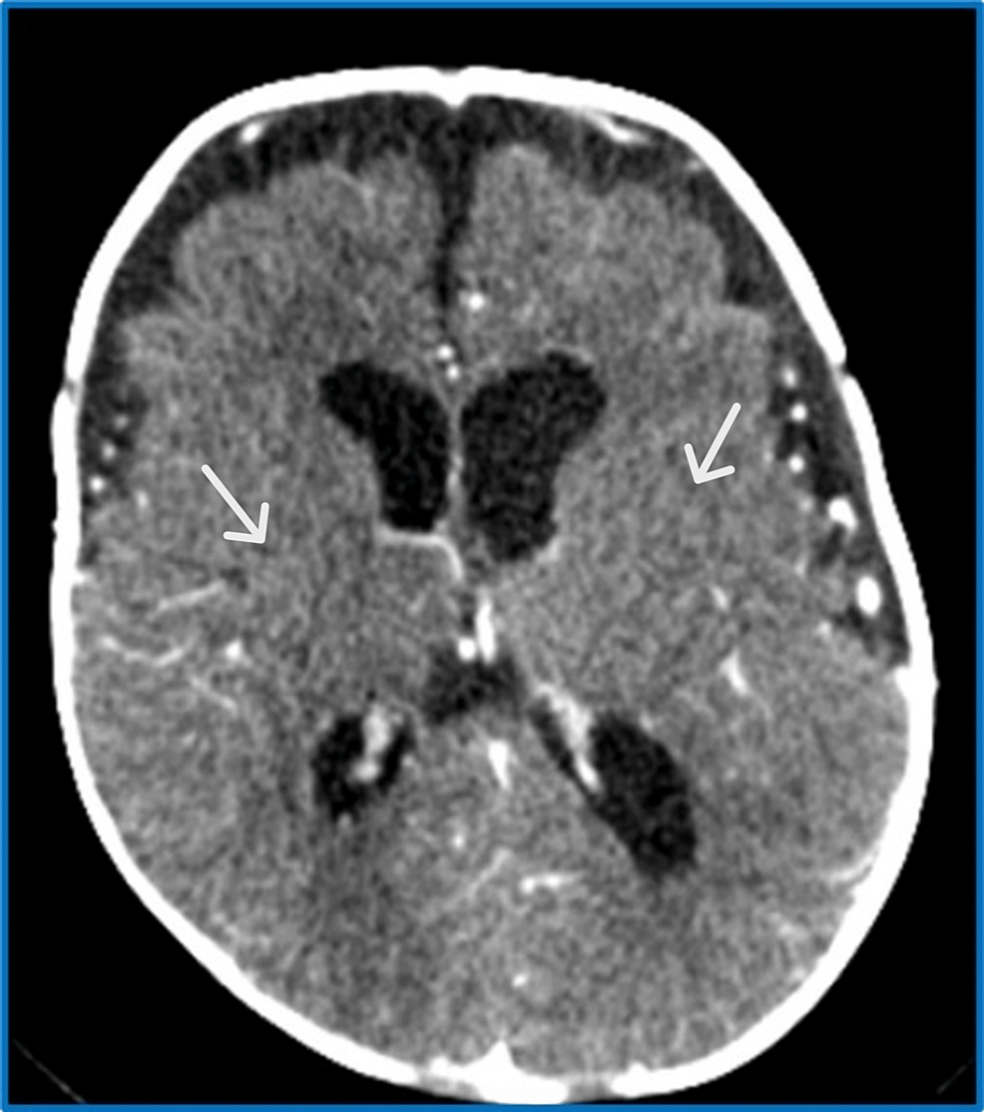
Brain CT on the day of admission showed signs of increased intracranial pressure (loss of gray matter and white matter differentiation marked by white arrows)

On examination, the anterior fontanelle was found to be full and open. The patient was administered a dose of diazepam and a loading dose of levetiracetam, along with paracetamol, ceftriaxone, vancomycin, acyclovir, and a normal saline bolus, intravenously. A CT of the brain was performed; it showed signs of increased intracranial pressure and no abscess formation (Figure 1).

The patient was extremely lethargic, and the rapid response team was activated to transfer the patient to the pediatric intensive care unit. He was diagnosed with meningoencephalitis with multiorgan dysfunction manifested in the form of hypotension that required inotropic support, respiratory failure, bone marrow suppression, and brain edema. Hence, the patient received mannitol and 3% sodium chloride intravenously and was intubated. The cerebrospinal fluid (CSF) and blood cultures confirmed the presence of gram-negative Bacilli, followed by the presence of nontyphoidal *Salmonellae* sensitive to meropenem, ceftriaxone, and trimethoprim/sulfamethoxazole. The rest of the CSF analysis revealed the following: white cell count of 153 cells/mm3 (normal range: 0-5 cells/mm3), red cell count of 33 cells/mm3 (normal range: 0-10 cells/mm3), glucose 0.1 mmol/l (normal range: 2.2-3.9 mmol/l), protein 2.058 g/L (normal range: 0.15-0.6 g/L), and the polymerase chain reaction (PCR) for meningitis yielded negative results. Accordingly, acyclovir and vancomycin were discontinued. The complete blood count (CBC) and C-reactive protein (CRP) are summarized in Table 1.

**Table 1:** Initial laboratory results

Parameters	Patient’s Results	Reference Range
White blood count	2.99 x 10^9^/L	(4.3-11.3) x 10^9^/L
Hemoglobin	8.4 g/dl	(9.5-14.5) g/dl
Mean corpuscular hemoglobin	24.9 pg	(25-35) pg
Mean corpuscular volume	78.7 fL	(74-108 ) fL
Hematocrit	26.6 %	(31-41) %
Platelet count	82 x 10^9^/L	(150-450) x 10^9^/L
C-reactive protein	292 mg/L	<3 mg/L

**Figure 2:**
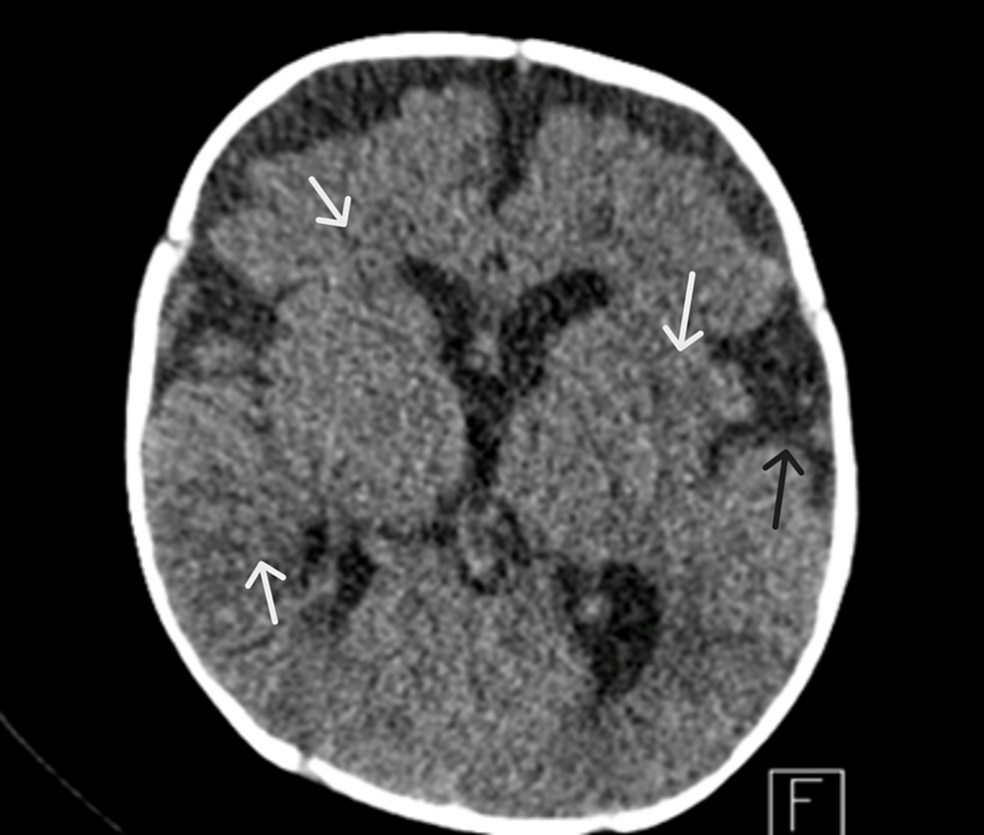
Repeat brain CT White arrows: Interval development of bilateral diffuse deep and periventricular white matter hypodensities; Black arrow: Minimal interval increases in the dilated extra-axial subarachnoid spaces, suggesting the presence of increased intracranial pressure.

On day 3 of admission, the patient tested positive for SARS-CoV-2 nucleic acid based on reverse transcription polymerase chain reaction (RT-PCR) testing in accordance with the WHO guidelines. On day 4 of admission, he developed two episodes of focal seizures that involved the right lower limb. The brain CT was repeated; it demonstrated interval development of bilateral diffuse deep and periventricular white matter hypodensities with minimal increases in the dilated extra-axial subarachnoid spaces that suggested increased intracranial pressure (Figure 2).

During the following days, the patient was tachypneic and desaturating, and chest X-ray images showed a right-sided pneumothorax. Accordingly, needle decompression was performed, and a right chest tube was inserted. Ventilation was then escalated to high-frequency mechanical ventilation, and nitric oxide was administered to manage acute respiratory distress syndrome (ARDS) secondary to pulmonary hypertension crises due to intrapulmonary shunting.

**Figure 3:**
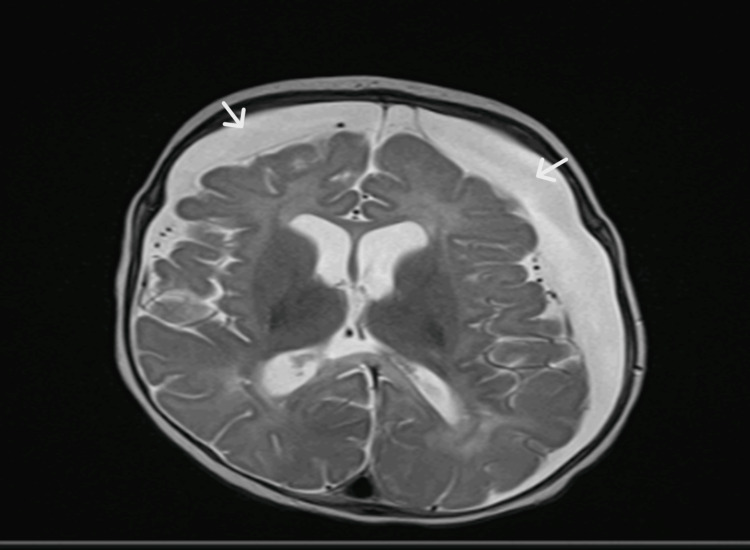
First brain MRI showing the bilateral subdural collections (white arrows) that caused a midline shift

**Figure 4:**
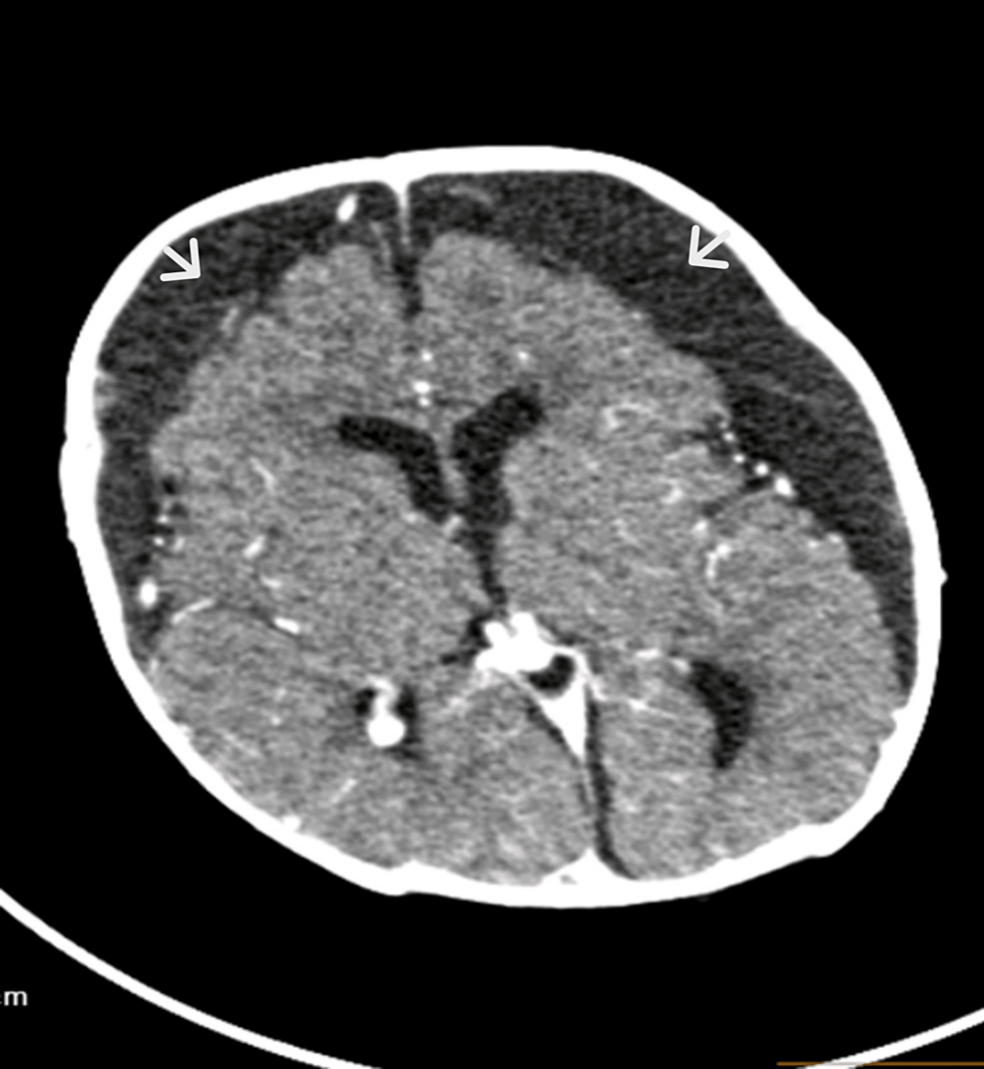
Repeat CT scan of the brain shows an interval increase in right subdural collections that measures 1.7 cm compared to the previous measurement of 1.1 cm (white arrows)

The MRI of the brain performed on day 19 of admission showed acute ischemic changes of the corpus callosum in compliance with the findings of hypoxic-ischemic encephalopathy and bilateral subdural collections (Figure 3). The patient was extubated successfully after 20 days of noninvasive mechanical ventilation. A few days later, he started to develop persistent spikes of fever, and a repeated CT scan of the brain showed an interval increase in the right subdural collections with a new measurement of 1.7 cm vs. the prior measurement in an MRI of 1.1 cm. Filing defects were noted along the right transverse sinus concerning thrombosis; accordingly, the patient was started on enoxaparin (Figure 4). The analysis of serial CSF is shown in Table 2, and serial other cultures are shown in Table 3.

**Table 2:** Serial CSF analysis PCR: Polymerase chain reaction, CSF: Cerebrospinal fluid

Day of admission	WBC cells/mm^3^	RBC cells/mm^3^	Protein g/l	Glucose g/l	Gram stain	Culture	PCR
1^st^	153	33	2.058	0.1	Salmonella spp.	Salmonella spp.	Negative
10^th^	76	33	0.962	3.3	Negative	Negative	Negative
12^th^	-	-	-		Negative	Negative	-

**Table 3:** Serial other cultures

Site of Culture	Day 1	Day 5	Day 7
Blood peripheral	Salmonella Spp	Negative	Negative
Blood central	-	Negative	Negative
Urine	No growth	No growth	No growth

One month after admission, the patient was transferred to the ward, and a nasal cannula was inserted for respiratory support. Brain and spine MRIs were repeated one week later; these showed signs of diffuse, extensive dural and leptomeningitis. A mild interval increase of the bilateral holohemispheric subdural collections, mass effect, and mildly communicating hydrocephalus were observed. Subtle diffuse leptomeningeal enhancement along the spinal cord related to the known meningitis was further noted.

Neurosurgeons who were involved in this case since the patient’s admission intervened; a burr-hole evacuation procedure of the right-side collection was performed, and an external ventricular drain (EVD) catheter was inserted in the subdural space. The culture result of the collection was negative, and PCR could not be performed owing to the lack of PCR reagents at that time. Imaging repeated one week after the EVD removal demonstrated a mild interval reduction of the bilateral subdural collection (the left side measurement was 2 cm as opposed to the previous measurement of 2.9 cm, and the right side was 1.5 cm in contrast to the previous measurement of 2 cm). The evacuation of the collection was followed by serial brain MRIs to monitor its size while the patient was kept on ceftriaxone in addition to weekly CBC and CRP examinations. The CRP levels (292 mg/L at presentation) reached 3.19 mg/L after two months.

**Figure 5:**
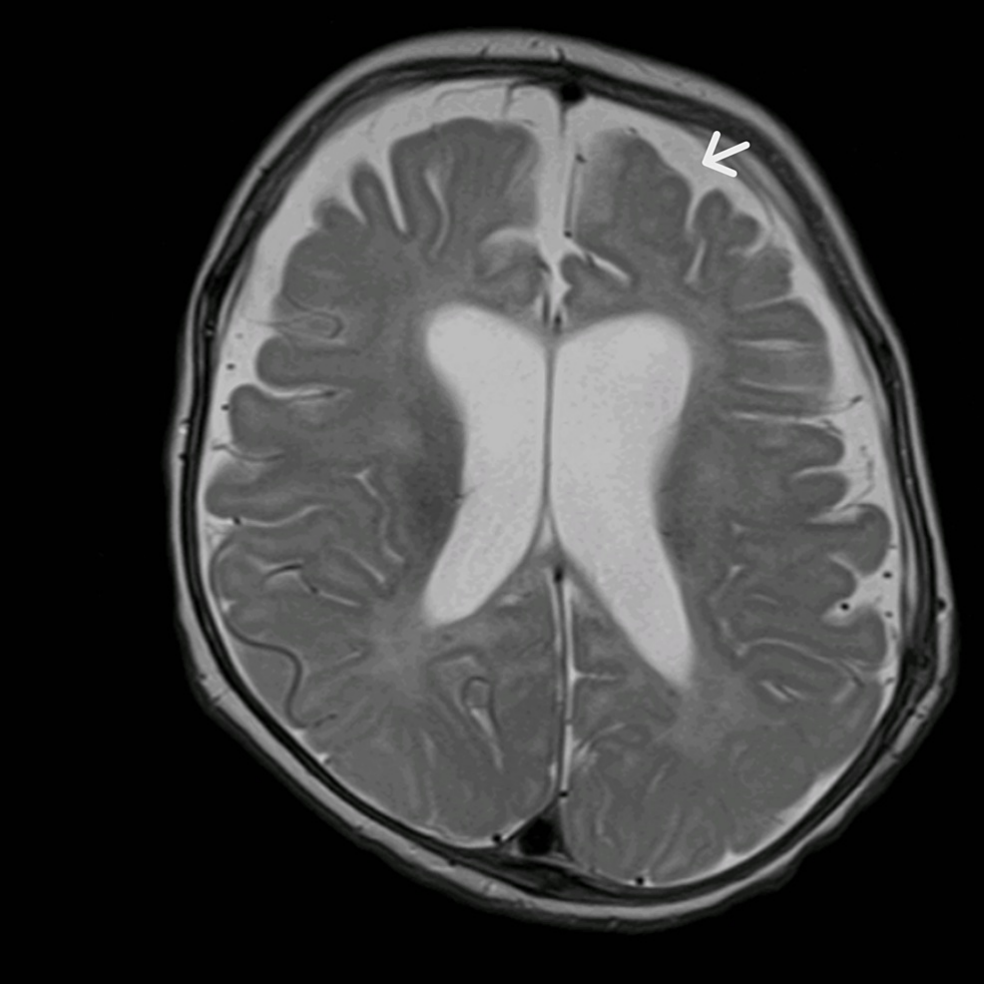
The final MRI of the brain performed prior to the discharge showed a decreased size of the previously observed left subdural fluid collection (white arrow)

The patient was discharged in April 2022 after the final brain MRI was performed (conducted on the day he was discharged). This scan showed a decreased size of the previously observed left subdural fluid collection, with a maximum thickness of 1.3 mm vs. the 3 mm previous measurement (Figure 5).

The patient has been followed up since his discharge on an outpatient basis in the General Pediatrics and Development Clinic. Furthermore, based on the patient’s clinical course, immunodeficiency was suspected. Therefore, an immunological workup was performed, which showed the following: IgG 2.46 g/l (normal range: 2.4-4.4); IgA 0.3 g/l (normal range: 0.27-0.86); IgM 0.27 g/l (normal range: 0.34 -1.14); CD3 1190 cells/mm3 (normal range: 2200-4100); and CD4 380 cells/mm3 (normal range: 1400-2800). Moreover, an invitae primary immunodeficiency panel test revealed one pathogenic variant in STXBP2 that is associated with autosomal recessive familial hemophagocytic lymphohistiocytosis.

## Discussion

The severity of COVID-19 was classified according to clinical features, laboratory investigations, and chest radiographic imaging into asymptomatic infection, mild, moderate, severe, and critical cases [3]. Severe cases showed an early onset of upper respiratory tract symptoms and gastrointestinal manifestations, and the disease usually progressed within approximately one week with shortness of breath and hypoxia symptoms, whereas in critical cases, children’s states may progress quickly due to acute respiratory distress syndrome or respiratory failure [3]. Further, they may develop multiorgan dysfunction syndrome [3]. A systemic review and meta-analysis of 5829 pediatric cases with COVID-19 concluded that severe cases under the age of one year accounted for 7% of pediatric cases, whereas critical ones represented 14% [3].

Adverse outcomes in COVID-19 patients are often due to bacterial co-infections [2]. In the literature, bacterial co-infection (community-acquired) is defined based on positive culture specimens acquired within 48 or 72 hours following admission [2,4]. Based on a meta-analysis, community-acquired COVID-19 has been identified in 3.5% of all cases, compared with secondary infections (hospital-acquired) identified in 14.3% of all cases [4,5]. The most common source of bacterial co-infection was pneumonia [2]. The most commonly reported organisms were gram-negative bacteria, mainly *Mycoplasma* species, followed by *Haemophilus influenzae* and *Pseudomonas aeruginosa* [4,5]. However, there was no clear mention of whether they were identified in secondary infections or co-infections, except in one study where *Staphylococcus aureus* followed by *Streptococcus pneumoniae* were the common pathogens in co-infections, unlike secondary infections wherein gram-negative bacteria were the dominant ones [5].

Risk factors for bacterial infections in moderate-to-critically ill patients with COVID-19 include critical illness at the time of presentation and the use of steroids [2]. To date, there is no published data regarding community-acquired bacteremia in children with COVID-19. Moreover, whether this type of bacteremia plays a role in determining the prognosis of SARS-CoV-2 infection or not remains unknown. In one study that included 836 adult patients with COVID-19, 17 out of 21 positive uncontaminated blood cultures were community-acquired infections [6]. There was a relative increase in the mortality risk of the patients with the presence of pathogens in their blood cultures, regardless of the source of infection, i.e., irrespective of whether it was community-acquired or hospital-acquired [6].

Nontyphoidal *Salmonellae* are bacterial pathogens that cause food-borne infections and are usually confined to the gastrointestinal tract in healthy children; however, several factors, including younger ages (specifically <1 year), underlying diseases, and immunosuppression, are risk factors for invasive disease [7]. *Salmonella* meningitis is a rare event, and infants aged <6 months have the highest risk of developing it. There are several associated neurological complications, such as hydrocephalus, subdural empyema, ventriculitis, cerebral abscesses, convulsive disorder, and coma. Furthermore, it is associated with high fatality rates [7].

## Conclusions

This is the first case report of COVID-19 in association with Salmonella bacteremia and meningitis. Whether the reason for this patient’s critical course was related to COVID-19 itself or to the co-infection with Salmonella is a valuable question. Additional studies have to be conducted about community-acquired bacteremia in children with COVID-19 to determine whether or not it affects overall mortality and morbidity.
